# Lack of access to chemotherapy for colon cancer: multiplicative disadvantage of being extremely poor, inadequately insured and African American

**DOI:** 10.1186/1472-6963-14-133

**Published:** 2014-03-22

**Authors:** Kevin M Gorey, Sundus Haji-Jama, Emma Bartfay, Isaac N Luginaah, Frances C Wright, Sindu M Kanjeekal

**Affiliations:** 1 School of Social Work, University of Windsor, 401 Sunset Avenue, Windsor, Ontario N9B, 3P4, Canada; 2 Faculty of Health Sciences, University of Ontario Institute of Technology, Oshawa, Ontario, Canada; 3 Department of Geography, University of Western Ontario, London, Ontario, Canada; 4 Sunnybrook Health Sciences Center and cross appointed to the Departments of Surgery and Health Policy, Management and Evaluation, University of Toronto, Toronto, Ontario, Canada; 5 Windsor Regional Cancer Center, Windsor, Ontario, Canada

**Keywords:** Health insurance, Uninsured, Underinsured, Poverty, Colon cancer, Chemotherapy, African American, Ethnicity, Health care reform, California, United States

## Abstract

**Background:**

Despite evidence of chemotherapy’s ability to cure or comfort those with colon cancer, nearly half of such Americans do not receive it. African Americans (AA) seem particularly disadvantaged. An ethnicity by poverty by health insurance interaction was hypothesized such that the multiplicative disadvantage of being extremely poor and inadequately insured is worse for AAs than for non-Hispanic white Americans (NHWA).

**Methods:**

California registry data were analyzed for 459 AAs and 3,001 NHWAs diagnosed with stage II to IV colon cancer between 1996 and 2000 and followed until 2011. Socioeconomic data from the 2000 census categorized neighborhoods: extremely poor (≥ 30% of households poor), middle (5-29% poor) and low poverty (< 5% poor). Participants were randomly selected from these poverty strata. Primary health insurers were Medicaid, Medicare, private or none. Chemotherapy rates were age and stage-adjusted and comparisons used standardized rate ratios (RR). Logistic and Cox regressions, respectively, modeled chemotherapy receipt and long term survival.

**Results:**

A significant 3-way ethnicity by poverty by health insurance interaction effect on chemotherapy receipt was observed. Among those who did not live in extremely poor neighborhoods and were adequately insured privately or by Medicare, chemotherapy rates did not differ significantly between AAs (37.7%) and NHWAs (39.5%). Among those who lived in extremely poor neighborhoods and were inadequately insured by Medicaid or uninsured, AAs (14.6%) were nearly 60% less likely to receive chemotherapy than were NHWAs (25.5%, RR = 0.41). When the 3-way interaction effect as well as the main effects of poverty, health insurance and chemotherapy was accounted for, survival rates of AAs and NHWAs were the same.

**Conclusions:**

The multiplicative barrier to colon cancer care that results from being extremely poor and inadequately insured is worse for AAs than it is for NHWAs. AAs are more prevalently poor, inadequately insured, and have fewer assets so they are probably less able to absorb the indirect and direct, but uncovered, costs of colon cancer care. Policy makers ought to be cognizant of these factors as they implement the Affordable Care Act and consider future health care reforms.

## Background

Chemotherapy provided after surgical resection is beneficial for many with non-localized colon cancer. Survival benefits are large for many with advanced, but non-metastasized, stage III disease and smaller, but still significant for some with stage II disease [1-3]. There are probably even small survival benefits of chemotherapy for people with metastasized, stage IV colon cancer, in addition to its clear palliative benefits [4]. Yet nearly half of all such people in the United States do not receive chemotherapy with the intention to either cure or comfort [5]. Moreover, chemotherapy access may be affected by social and economic characteristics such as race or ethnicity, and income and health insurance adequacy. African Americans (AA), who are also poorer and less adequately insured, on average, than non-Hispanic white Americans (NHWA), seem particularly disadvantaged [6,7]. They are less likely to receive adjuvant chemotherapy and this can explain much of their survival disadvantage [5,8-10].

Social and health care systemic factors seem most implicated in this racial divide for the following reasons. First, clinical studies of equal-access health care systems within the US have consistently observed similar chemotherapy and survival rates among AAs and NHWAs with colon cancer [11-14]. Second, colon cancer chemotherapy rates seem to be much higher in Canada than in the US [15,16]. Third, recent population-based studies of colon cancer care in California and Ontario suggested that people of color, including black people of various ethnic backgrounds, with colon cancer are much better served in California [16-18]. Moreover, health insurance inadequacies in the US versus universal coverage of medically necessary care in Canada largely explained the between-country divide. Being uninsured or insured by Medicaid has been consistently found to be less adequate than being insured by Medicare or by a private insurer [5,17,18]. Fourth, this field’s research syntheses allow for the conclusion that socioeconomic status (SES) and treatment differences largely explain observed AA-NHWA survival differences [5,8-10]. The fact that they have largely, but not completely accounted for racial differences with social-systemic factors suggests the plausibility of at least one of their causes being biological. For example, AA and NHWA patients may differ on oncogene-based tumor characteristics that affect survival directly or indirectly, through their effects upon clinicians’ decisions to prescribe chemotherapies. Modest equivocal differences between racialized groups on colon tumor grade [8], however, suggest that, though possible, this explanation for the differential uptake of chemotherapy and survival differences between AAs and NHWAs is improbable.

In attempting to account for SES, this field’s population-based studies have typically used census data to define low-income neighborhoods. However, their lowest income neighborhoods only ranged from 10% to 20% poor. They therefore had limited power to study colon cancer care among the “truly disadvantaged” [19] who live, for example, in America’s poorest neighborhoods where 30% to 40% or more of households have incomes below the poverty line [19-21]. Consequently, they probably did not account for residual confounding. That is, their AA participants probably had substantially lower incomes than their NHWA counterparts, even in the lowest income neighborhoods studied [22,23]. This field also seems limited by its focus on the mere main effects of race/ethnicity, SES and health insurance. Recognizing that it is important to analyze different racial/ethnic minority groups uniquely, our previous research on breast cancer care among Mexican American women leads us to anticipate complex interactions of ethnicity, poverty and health insurance status [24-26]. We were recently presented with a serendipitous opportunity to systematically replicate that notion among AAs with colon cancer.

We oversampled people with colon or breast cancer in the highest poverty neighborhoods of California for other primary studies [17,18,24]. This necessarily meant that we also oversampled AAs. Secondary to our original study’s intentions we had the opportunity to compare the chemotherapy and survival experiences of AAs and NHWAs with colon cancer while providing more control for residual confounding by SES than previous studies had. This field’s historical-theoretical context strongly suggests multiplicative, rather than additive disadvantages of being poor and inadequately insured among AAs. That is, the combined effects of being poor and of being uninsured or underinsured on colon cancer care are probably worse for AAs than for NHWAs. Therefore, we hypothesize a 3-way ethnicity by poverty by health insurance interaction on the receipt of chemotherapy. Secondarily, we hypothesize that this complex interaction, along with the main effects of poverty, health insurance adequacy and treatment access, will now *completely* explain any observed AA-NHWA survival differences.

## Methods

Six thousand, three hundred people who were diagnosed with colon cancer between 1996 and 2000 were randomly selected from the California Cancer Registry (CCR) that was joined to the 2000 census by census tracts [17,18,27]. The original sample was also stratified by SES (extremely poor neighborhoods where 30% or more of the households were poor, 5% to 29% or less than 5% poor) and place (large or smaller urban or rural). This secondary analysis excluded localized, stage I cancers for which chemotherapy is not indicated as well as people of other racial or ethnic backgrounds [28,29]. This study then analyzed colon cancer care among 459 AAs (also non-Hispanic) and 3,001 NHWAs with stage II to IV disease.

A logistic regression model was used to test the 3-way ethnicity (AA vs. NHWA) by poverty (extremely poor or not) by health insurance adequacy (uninsured or Medicaid insured vs. privately or Medicare insured) interaction in predicting binary chemotherapy receipt [30]. Odds ratios (OR) and 95% confidence intervals (CI) were estimated from regression statistics. AA study participants were younger (*M* = 67.4, *SD* = 13.9), on average, than the NHWA participants (*M* = 70.9, *SD* = 13.0), *F* (1, 3,458) = 28.39, *p* < .05. Therefore, all rates were internally age-adjusted and reported as percentages (rates per 100). Chemotherapy rates were also stage-adjusted as clinical indication and prescription rates differ significantly for stage II, III and IV disease. Then standardized rate ratios (RR) were reported for critical between-group comparisons with 95% CIs derived from the Mantel-Haenszel χ^2^ test. Standardized rate difference (RD) indices were also used to further aid the interpretation of practical-clinical significance. The hypothesis that the main and *interacting* effects of ethnicity, poverty, health insurance and chemotherapy access would completely explain any observed AA-NHWA survival difference was tested with unadjusted and adjusted Cox proportional hazards regression models [31]. All participants were minimally followed for 10 years, from the date of their diagnosis until January 1, 2011. Hazard ratios (HR) and 95% CIs were estimated from regression statistics. Other methodological details have been reported [17,18,27]. This study was reviewed and cleared by the University of Windsor research ethics board.

## Results

### Description of AA and NHWA samples

Unadjusted descriptive profiles of the AA and NHWA samples are displayed in Table 1. The statistically significant comparisons seem consistent with existing knowledge. AAs were much more likely to live in high poverty neighborhoods and seemed more likely to be either uninsured or to be insured by Medicaid. Interestingly, oversampling from poor neighborhoods seems to have provided ample control for SES and biological characteristics. Median annual household incomes among extremely poor AAs ($22,600) and NHWAs ($23,650), though significantly different in a statistical sense, were actually quite similar. Overall, the two ethnic groups did not differ significantly on either tumor grade or on stage of disease at diagnoses. AAs seemed somewhat less likely to receive chemotherapy.

**Table 1:** Socio-demographic and clinical characteristics of African American and non-Hispanic white American colon cancer patients in California, 1996-2000

	**African American, No. (%)**		**Non-Hispanic White, No. (%)**	
Age at diagnosis,^**^ y				
25 - 59	129	28.1	563	18.8
60 - 69	99	21.6	629	21.0
70 - 79	138	30.1	980	32.7
≥ 80	93	20.3	829	27.6
Women^**^	275	59.9	1,574	52.4
Neighborhood poverty prevalence,^**^%				
< 5	31	6.8	1,240	41.3
5 - 29	76	16.6	1,131	37.7
≥ 30^1^	352	76.7	630	21.0
Primary health insurers^**^				
Private	194	42.3	1,409	47.0
Medicare	201	43.8	1,391	46.4
Medicaid	36	7.8	72	2.4
Uninsured	28	6.1	129	4.3
Stage at diagnosis				
II	184	40.1	1,270	42.3
III	132	28.8	927	30.9
IV	143	31.2	804	26.8
Tumor grade				
I	31	7.4	192	6.8
II	286	68.4	1,863	66.4
III or IV^2^	101	24.2	752	26.8
Missing data^*^	41	8.9	194	6.5
Examined 12 or more RLN^3^	124	40.4	939	44.1
Missing data	9	2.8	67	3.0
Received chemotherapy^*^	145	31.7	1,124	37.6
Missing data	2	0.4	9	0.3

*Note. RLN*, Regional lymph nodes.

^1^Median annual household income for extremely poor AA ($22,600) and NHWA ($23,650) subsamples; median test [32], χ^2^ (1, *N* = 982) = 5.51, *p* < .05.

^2^Only 23 (0.7%) of the tumors were undifferentiated or grade IV.

^3^Stage IV metastasized disease excluded.

^*^p < .10 and ^**^p < .05 for between-ethnic group difference (χ^2^ test).

Age-adjusted comparisons served to clarify the socioeconomic divide between AAs and NHWAs. AA patients (77.3%) were nearly four times as likely as NHWA patients (20.9%) to live in extremely poor neighborhoods (RR = 3.70, 95% CI 3.33, 4.11), they were nearly twice as likely to be either uninsured to Medicaid-insured (12.0% vs. 6.9%, RR = 1.74, 95% CI 1.31, 2.31) while they were 17% less likely to be privately insured (39.4% vs. 47.5%, RR = 0.83, 95% CI 0.74, 0.93). While one of every nine AA study participants (11.3%) suffered the multiplicative disadvantage of living in an extremely poor neighborhood and being inadequately insured, only one of every 36 NHWA participants was so disadvantaged (RR = 4.03, 95% CI 2.97, 5.48). Finally, the AA patients were 26% less likely to receive chemotherapy (age- and stage-adjusted rates of 28.2% vs. 38.2%, RR = 0.74, 95% CI 0.63, 0.85). It should be noted that nearly all (99.0%) of the patients with stage II or III disease received surgical resections. Fewer with stage IV disease had surgery, and among them fewer of the AA (60.1%) than NHWA (73.1%) patients were so treated (RR = 0.82, 95% CI 0.73, 0.93). However, surgery refusal rates (5.5%) were identical for the AAs and NHWAs with metastasized disease.

### Ethnicity by poverty by health insurance interaction

As hypothesized, a significant 3-way ethnicity by poverty by health insurance interaction was detected (OR = 1.46, 95% CI 1.04, 2.06) with an age, stage and grade-adjusted logistic regression on chemotherapy receipt that included the well-known main disadvantaging effects of being AA (OR = 0.64, 95% CI 0.48, 0.84), extremely poor (OR = 0.70, 95% CI 0.58, 0.85) and inadequately insured (OR = 0.60, 95% CI 0.40, 0.89). Neither main or interacting effects of gender nor the number of region lymph nodes examined entered the model. As interpretation of the 3-way interaction effect’s point-estimate (OR = 1.46) is not intuitive, the interaction is practically depicted in Table 2. At the top of the table it can be seen that among those who did not live in extremely poor neighborhoods and were adequately insured, chemotherapy rates did not differ significantly between AAs and NHWAs. Moving down the table one sees that among those with one of two disadvantages, AAs were 20% less likely to receive chemotherapy (RR = 0.80). And at the bottom of the table we see that among the multiply disadvantaged, the disadvantaging effect on chemotherapy access appeared multiplicative. Among them AAs were nearly 60% less likely to receive chemotherapy than were NHWAs (RR = 0.41). One should note that chemotherapy refusal rates did not differ between these, most disadvantaged, AA (5.9%) and NHWA (6.3%) patients; χ^2^ (1, *N* = 130) = 0.01, *p* = .92.

**Table 2:** Effects of the interaction of ethnicity, neighborhood poverty and health insurance on chemotherapy receipt in California, 1996-2000

					**NHWA-AA**
	**No.**^**1**^	**Rate**	**RR**^**2**^	**(95% CI)**	**Chemotherapy RD**
< 30% Poor & Adequately Insured					
Non-Hispanic white American	2,249	.395	1.00		
African American	94	.377	0.95	(0.75, 1.20)	1.8%
Intermediate Groups^3^					
Non-Hispanic white American	673	.345	1.00		
African American	314	.277	0.80	(0.65, 0.98)	6.8%
≥ 30% Poor & Inadequately Insured					
Non-Hispanic white American	79	.352	1.00		
African American	51	.146	0.41	(0.22, 0.78)	20.6%

*Notes. NHWA*, Non-Hispanic white American; *AA*, African American; *RR*, Standardized rate ratio; *RD*, Standardized rate difference; *CI*, Confidence interval. All rates were directly age and stage-adjusted using this study’s combined AA-NHWA population of cases as the standard.

^1^Number of incident colon cancer cases.

^2^A rate ratio of 1.00 was the within-ethnic group baseline.

^3^Included those who lived in extremely poor neighborhoods, but were adequately insured or those who lived in less poor neighborhoods, but were inadequately insured.

The multiplicative disadvantage of extremely poor and inadequately insured AAs is depicted in another, perhaps more clinically telling way, in the table’s right column. The NHWA-AA RD on chemotherapy receipt was 1.8% among the most advantaged group. While the RD of 20.6% among the most disadvantaged group was more than a 10-fold multiple of that baseline difference. Such seems indicative of a huge chemotherapy access barrier. The support we found for our secondary hypothesis strongly suggests though that it can be effectively remediated. An age, gender, stage and grade-adjusted Cox regression on survival found that the AA patients were much more likely to die over the 10 year follow-up period (HR = 1.26, 95% CI 1.13, 1.42). When another model was run in which the main effects of poverty, health insurance and chemotherapy as well as the interaction effect of ethnicity-poverty-health insurance were entered, AAs and NHWAs experienced identical risks of death (HR = 1.01, 95% CI 0.71, 1.43).

## Discussion

First, this field’s established ethnicity-outcome relationships were systematically replicated among historical cohorts of AAs and NHWAs with non-localized colon cancer. This study’s AA participants were about 25% less likely to receive chemotherapy and about 25% more likely to die over the 10 years following their diagnosis than were its NHWA participants. Next, this field’s well-known main predictive effects were replicated. AAs, those who lived in extremely poor neighborhoods and those who were inadequately insured were all significantly less likely than NHWAs, the less poor or the adequately insured to receive chemotherapy and so more likely to die sooner. Then an ethnicity by poverty by health insurance interaction on chemotherapy receipt was discovered. This study’s central original finding, the 3-way interaction is evidence of the multiplicative disadvantage experienced by extremely poor and inadequately insured AAs seeking colon cancer care. Among relatively advantaged people, AA and NHWA chemotherapy rates differ little, if at all (< 2%). However, among the multiply disadvantaged extremely poor and inadequately insured, the AA-NHWA chemotherapy RD (> 20%) could, we think, be fairly characterized as huge in terms of both its clinical and human significance. Finally, controlling for residual SES confounding by oversampling from extremely poor neighborhoods and further controlling for demographic and biological differences through mathematical modeling, we discovered that the main and interacting effects of poverty, health insurance adequacy and treatment access can completely account for the typically observed AA-NHWA survival differences. After we accounted for such social forces, the survival experiences of the AA and NHWA colon cancer patients we studied were essentially identical.

Two-way poverty by health insurance interactions have previously been observed in studies of breast and colon cancer care among NHWAs [18,24,25]. The beneficial effects of health insurance were observed to be strongest in low poverty neighborhoods. It was theorized that the effectiveness of health insurance programs was positively impacted by the availability of other key resources. In more affluent neighborhoods, where social and economic capital abounds, most people with cancer seemed able to absorb the indirect (time lost from work, recuperation, transportation and others) and direct, but additional uncovered, out-of-pocket costs of care (co-insurance and co-payments). Within high poverty neighborhoods on the other hand, relatively lacking in such capital reserves, health insurance programs seemed to be much less effective. Such extremely poor people were probably much less able to pick-up the co-insurance costs and co-payments that are prevalent in American cancer care. The 3-way ethnicity-poverty-health insurance interaction observed in this study may be thought of as an extension of the previously observed 2-way poverty-health insurance interaction. It strongly suggests that the 2-way interaction’s effect is stronger for AAs. Their relative lack of capital reserves could explain this differential ethnic effect [33,34]. We saw that this study’s oversampling from extremely poor neighborhoods seemed to fairly well control AA-NHWA differences on depth of impoverishment [22,23], at least in terms of their annual household incomes that only differed by about $1,000. However, a contemporaneous analysis found that among the poorest households in America, NHWAs typically held $10,000 worth of equity, while AAs typically had no assets at all [7]. It seems probable that their lack of capital reserves operates to further potentiate the disadvantages already experienced by extremely poor and inadequately insured AAs as they try to purchase chemotherapeutic and other colon cancer care services.

### Practical—clinical and policy—significance

Approximately 13,300 AAs are diagnosed with colon cancer each year [35,36]. Unfortunately, they quite commonly live in poor, often extremely poor, neighborhoods (one of every four or five households) and are also commonly uninsured or underinsured (one of every three or four households) [6,20]. Applying this study’s chemotherapy estimates to these population parameters and social exposures we estimate that each year slightly more than 1,500 AAs with colon cancer are treated less optimally than are NHWAs of similar ages with colon cancers of similar stages and grades [37]. That represents 22,500 relatively undertreated AAs with colon cancer during the 15 years that preceded passage of the Patient Protection and Affordable Care Act, so-called Obamacare. This striking inequity is probably only the tip of the iceberg of AA disadvantages as colon cancer accounts for less than 3% of the burden of disease in the US [38]. One should hope that as Obamacare is rolled out and provides millions more Americans with health insurance that it will also diminish the racial and ethnic divides that presently exist in American health care. Such will probably depend largely upon the adequacy of the new health insurances it provides. Health care reformers need to account for the fact that even covered health care, especially for such diseases as colon cancer [39], has myriad out-of-pocket costs. Health insurance programs that serve to minimize such costs will necessarily be more effective than those that do not.

### Potential limitations

This study could have been limited by incomplete information on chemotherapy. Because chemotherapy is most often received as an outpatient it can be more challenging for cancer registries to survey. For the following reasons we think it not a potent alternative explanation. First, the CCR has been demonstrated to be mostly complete (81% to 84%) on chemotherapy and errors have been demonstrated not to differ significantly by race/ethnicity or income [40,41]. Second, missing chemotherapy data was modest and did not differ between this study’s AA and NHWA samples. Third, analyses of health insurance, surgeries and survival were unlikely to have been affected [41-44] and modest errors very likely did not differ by socioeconomic factors [42]. Such nondifferential errors suggest that any bias would probably have been toward the null [45,46]. That is, the magnitude of this study’s observed AA disadvantages may, in fact, be underestimates of the truth.

This study’s findings could also have been confounded by comorbid differences between its AA and NHWA samples. The CCR did not code comorbidities that are well known to be associated with socioeconomic factors, colon cancer care and survival [5]. However, AAs and NHWAs with similar disease stages were compared and through mathematical modeling, essentially matched on a proxy of cancer virulence, tumor grade, and on two strong correlates of other chronic diseases, age and poverty. Therefore, the two groups seemed to be quite similarly diseased, making comorbid alternative explanations unlikely.

## Conclusions

Overall, AAs with non-localized colon cancer are 25% less likely to receive indicated chemotherapy than are NHWAs with similar colon cancers. Among the extremely poor and inadequately insured AAs are 60% less likely than NHWAs to receive such care. Ethnicity, poverty and health insurance status appear to interact in such a way that the multiplicative barriers to care of being extremely poor and inadequately insured are worse for AAs than they are for NHWAs. AAs are more prevalently poor and inadequately insured, but even when depth of income-based poverty is controlled, they still have substantially fewer assets than NHWAs so they are probably much less able to absorb the indirect and direct, but uncovered, costs of colon cancer care. Policy makers ought to be cognizant of these factors as they roll out Obamacare and consider future reforms of health care in America.

## Competing interests

The authors declare that they have no competing interests.

## Authors’ contributions

KMG conceptualized the study, led the study design and writing and supervised the analysis. SH performed the analysis and assisted with study design, data interpretation and writing. INL, EB, FCW and SMK assisted with study design, data interpretation and writing. KMG and INL also obtained funding. All authors read and approved the final manuscript.

## Pre-publication history

The pre-publication history for this paper can be accessed here:

http://www.biomedcentral.com/1472-6963/14/133/prepub
